# Mast Cell Responses to Viruses and Pathogen Products

**DOI:** 10.3390/ijms20174241

**Published:** 2019-08-30

**Authors:** Jean S. Marshall, Liliana Portales-Cervantes, Edwin Leong

**Affiliations:** 1 Dalhousie Human Immunology and Inflammation Group, Department of Microbiology & Immunology, Dalhousie University, Halifax, NS B3H 4H7, Canada; 2 Dalhousie Human Immunology and Inflammation Group, Department of Pathology, Dalhousie University, Halifax, NS B3H 4H7, Canada

**Keywords:** mast cell, asthma, infection, interferon, NK cell, chemokine, immunotherapy, oncolytic

## Abstract

Mast cells are well accepted as important sentinel cells for host defence against selected pathogens. Their location at mucosal surfaces and ability to mobilize multiple aspects of early immune responses makes them critical contributors to effective immunity in several experimental settings. However, the interactions of mast cells with viruses and pathogen products are complex and can have both detrimental and positive impacts. There is substantial evidence for mast cell mobilization and activation of effector cells and mobilization of dendritic cells following viral challenge. These cells are a major and under-appreciated local source of type I and III interferons following viral challenge. However, mast cells have also been implicated in inappropriate inflammatory responses, long term fibrosis, and vascular leakage associated with viral infections. Progress in combating infection and boosting effective immunity requires a better understanding of mast cell responses to viral infection and the pathogen products and receptors we can employ to modify such responses. In this review, we outline some of the key known responses of mast cells to viral infection and their major responses to pathogen products. We have placed an emphasis on data obtained from human mast cells and aim to provide a framework for considering the complex interactions between mast cells and pathogens with a view to exploiting this knowledge therapeutically. Long-lived resident mast cells and their responses to viruses and pathogen products provide excellent opportunities to modify local immune responses that remain to be fully exploited in cancer immunotherapy, vaccination, and treatment of infectious diseases.

## 1. Introduction 

Mast cells (MC) are strategically placed at sites that interface with our external environment such as the skin, lung, and intestines. Within such tissues they are predominately below the epithelial layer and closely associated with blood vessels This location allows them to act as sentinels for tissue damage and pathogen invasion. It also places them close to other sentinel cells, such as dendritic cells. The association between MC and blood vessels is optimal to enhance the rapid recruitment of effector cells out of the bloodstream and into neighboring tissues. This process is facilitated by the MC’s rapid production of cytokine mediators such as TNF and IL-1β that activate endothelium, lipid mediators that facilitate vasodilatation, as well as a range of chemokines that promote the selective recruitment of specific subsets of effector cells. In this chapter, we will explore MC responses, focusing on those of human origin, to common viruses and pathogen products. We aim to highlight a range of MC–pathogen interactions, recognizing that it is impossible to include all the very important contributions to this field. Understanding effective host responses to infection is also critical for defining MC activation signals and responses by which immunity could be enhanced or inhibited in disease settings.

## 2. Historical Studies of MC–Virus and MC–Pathogen Product Interactions

Historically, the focus has been on the role of MCs in allergic disease. Interactions of MCs with most viral pathogens and pathogen products were not evaluated in detail until relatively modern times. Although not a topic for this review, substantial definition of the roles for MCs in parasitic diseases provided many of the tools needed to examine MCs in other infection settings. Initial studies often focused on the ability of pathogen products to induce degranulation. Several bacterial, fungal, and parasitic products were shown to either induce MC lysis or act through G-protein-coupled or Fc receptors, directly or indirectly to induce degranulation. The recognition that MCs could produce cytokines without the necessity for degranulation in response to bacterial products [1,2] opened up this area to include consideration of complex roles for MCs under circumstances where degranulation and an impact of MC stabilizers was not observed. 

Early studies of MC interactions with viruses focused on their ability to respond to the Sendai virus through granule release [3,4] and their immortalization using Harvey sarcoma virus, Kirsten sarcoma virus, and Abelson murine leukemia viruses [5,6,7]. However, even in these early days, there was a recognition that MCs may play a key role in inflammatory responses to viral infections in certain contexts, such as Sindbis virus infection of the central nervous system [8]. Many studies focused on the ability of MCs to become infected with common viruses and release classical MC mediators such as histamine and leukotrienes. These included studies of Para-influenza virus [9] and many others to be discussed below. More recently, a complex picture of MCs during infection has emerged, whereby they may promote effective immunity to infection under some circumstances, but also have the potential to contribute to tissue damage and impair vascular integrity, especially upon secondary infection. Important interactions observed in vitro and in vivo for a number of viruses including respiratory syncytial virus (RSV), rhinovirus (RV), reovirus, dengue virus (DENV), human immunodeficiency virus (HIV) and influenza, are noted in Table 1 and Table 2. 

## 3. Mast Cells and Mosquito-Borne Viruses

MCs respond to the bites of various insects including ticks and mosquitos. Indeed, mosquito saliva has been shown to have a number of impacts on MCs. Human in vitro systems and mouse models which include natural mosquito bites have provided evidence of mosquito bite-induced lymph node hypertrophy as well as demonstrations of mosquito-induced local immune suppression [34,35,36].

Studies of DENV initially demonstrated that human MC and MC lines produce a number of cytokines and chemokines following infection [37,38]. Infection was associated with high levels of MC apoptosis, and there is evidence of RNA sensors being involved in the initiation of chemokine responses [15,39]. Human skin MCs have also been shown to be infected in the context of mosquito-borne infection, with some evidence for infectious DENV associated with MC granules [13]. In vitro infection of human MCs with dengue was highly antibody-dependent and occurred through an FcγRII-dependent mechanism [40]. This could suggest that the major contribution of MCs in response to human DENV infection might be in the context of secondary infections that are associated with more severe disease. Consistent with this observation, clinical studies have revealed evidence of MC degranulation associated with dengue hemorrhagic disease and dengue shock syndrome [41,42,43]. Most recently, tryptase release, which is associated with severe forms of dengue disease, has been associated with endothelial barrier dysfunction [44]. In vitro, DENV infection of MCs leads to the production of other mediators that induce endothelial activation, such as IL-1β, together with chemokines capable of recruiting a variety of inflammatory and effector cells [15,45]. It is likely that multiple MC-dependent processes contribute to enhanced vascular leakage in severe disease. In the complex setting of secondary infection, other stimuli, such as complement components and Fc receptor cross-linking, may also play a role in activating MCs in response to local or disseminated infection. Studies in mice have suggested that a very high-dose DENV challenge can induce MC degranulation and mediator responses directly, without the necessity for antibody-dependent enhancement. They also demonstrate MC-dependent elicitation of an early innate response and subsequent acquired immunity [46]. MCs contribute to a response to infection that involves multiple cell types. In addition to MC endothelial cell interactions, the interface between dengue virus, monocytes and macrophages and MCs may be of particular importance [47]. Notably, MC infection with hantavirus has also been associated with a similar mediator response that may contribute to vascular dysfunction [48].

The Zika virus has similarities with DENV and is carried by the same mosquito vector. The ability of MCs to become infected with Zika and promote an immune or inflammatory response is an important area of current studies but few published studies are yet available. However, given the many similarities between Zika and DENV, both (+) RNA flaviviruses, it is likely that some shared responses and mechanisms to evade infection are in place. Histamine, IL-9, and a Th2 response have been clearly implicated in Zika disease pathology [49].

## 4. Responses to Respiratory Viruses 

Responses of MCs to respiratory virus infection are of interest in view of the association between allergic asthma exacerbations and viral infection. Early studies of bovine respiratory syncytial virus models demonstrated MC degranulation associated with infection in vivo [50,51]. This was followed by evidence of MC activation including both degranulation and lipid mediator release in wheezing human infants associated with RSV infection [52], along with suggestions of an important role for virus specific IgE in some cases [53]. More direct studies of interactions between RSV and MCs paint a slightly different picture. In the absence of virus-specific IgE, complement activation and other factors, which may have a role in vivo, RSV was found to have limited ability to induce degranulation or leukotriene generation. RSV also demonstrated limited transcription of viral products in human primary MCs. However, contact between RSV and human MCs induced substantial chemokine production as well as the production of type I interferons (IFNs) [16].

The impact of influenza and parainfluenza viruses on MC activation has also been a subject of considerable scrutiny. Early studies demonstrated mediator release by parainfluenza-treated calf and rodent MCs [9,54]. Studies in Brown Norway rats confirmed that in this strain, with higher numbers of MCs resident in airways tissues and a Th2 predisposition, there was evidence of MC hyperplasia, MC activation, and more severe airway inflammation [55,56]. In 2013, two important studies of the response of MCs to influenza A virus (IAV) infection were published. Graham et al. described a role for MCs in the response to IAV in vivo, although they were using an older c-kit-dependent MC deficiency model. This group also showed both MC degranulation and production of cytokines and chemokines by distinct mechanisms using rodent systems. Marcet et al., using human MC lines, demonstrated limited evidence of productive IAV replication, and again evidence of cytokine and chemokine production, as well as type I IFN production [57]. 

RV is possibly the most well-characterized infection, closely linked to asthma exacerbations. Similar to other respiratory viruses, it was also initially examined for impacts on classical MC mediator release and leukotriene generation [12,58]. Both human MC lines and cord blood-derived MCs support viral replication and produce a range of IFNs in response to RV infection [11]. Detailed recent studies of human MC lines have revealed complex responses which include some lactate dehydrogenase release and the production of several cytokines and chemokines, including CXCL8 and TNF [10]. Similar to DENV [39], RV also induces apoptosis in human MC lines [10]. Notably, RV infection of both primary MCs and MC lines is limited by pre-exposure of the cells to IFNβ [11]. This latter finding is in keeping with evidence that IFNβ production is deficient in asthmatics who show increased susceptibility to respiratory viral infection. Taken together, these findings suggest a role for MCs in enhancing asthma exacerbations and having a proinflammatory impact in response to RV.

In each of these settings, the human MC shows evidence of a response that is consistent with promoting early host defence, when in contact with a number of important respiratory viruses, which presumably would occur predominantly once infection has breached the epithelial barrier. MCs themselves are resistant to becoming productively infected with influenza or RSV but have a protective response that includes the production of cytokines and chemokines promoting the recruitment of antiviral effector cells. In addition, human MCs produce substantial amounts of type I IFNs which would promote a local antiviral response and resistance to infection. Of course, in a more disseminated infection, especially in situations where MC numbers were elevated, these responses could also contribute to potentially damaging inflammation. In the case of RV, MCs themselves are productively infected, but retain the ability to initiate host defence processes. Together with the potential impact of antiviral antibody responses (via both complement products and Fc receptor-mediated activation), it would be expected that the impacts of MCs on infection and the potential for a more damaging inflammatory response would be heightened upon secondary or subsequent infection with related viruses, although few studies have examined these issues directly.

## 5. Mast Cell Responses to HIV

MCs are found in substantial numbers at most mucosal surfaces, including the urinogenital and gastrointestinal tract. MC progenitors in the blood express low levels of CD4, CCR3, CXCR4, and CCR5. This profile led to their consideration as a target for HIV infection. Two early reports demonstrated infection of MC precursors and defined both a role for CCR5 and a propensity for infection with M tropic virus strains [59,60]. In one of these reports, it was suggested that circulating MC precursors from allergic individuals might be more susceptible to infection [60]. IL-16 was shown to limit the susceptibility of MC precursors to infection with M tropic virus [21]. It was also suggested from early work that HIV-1 gp120 acted as a viral superantigen. It was observed that HIV gp120 interacted with the heavy chain, variable 3 region of IgE, to induce cytokine release from FcεRI-positive cells. Possibly of greater importance clinically, was the suggestion that human MCs have the potential to act as a viral reservoir. Human MCs in vitro that were positive for proviral DNA showed evidence of productive HIV-1 infection following activation via Toll-like receptors [61]. In support of a potential viral reservoir role for MCs, HIV-infected women were found to have placental tissue MCs with inducible infectious HIV, despite antiretroviral therapy [62]. These observations proved controversial [63] and may be more important in the context of pregnancy associated immune suppression. The potential for MCs to aid in the spread of HIV has also been examined and both the transfer of virus to T cells by MC-dependent mechanisms and the recruitment of MCs by HIV-derived Nef protein has been reported [20,64]. Although all of these processes are of interest to understanding disease pathology and help us to understand the roles of MCs in infection, there remains little direct evidence for a critical clinical role for MCs in the susceptibility to or course of HIV infection.

## 6. Mast Cell Responses to Hepatitis Viruses

Although MCs are associated with blood vessels in most tissues, including the liver, the normal hepatic tissues are not MC-rich. However, following viral infection several reports suggest that MC numbers increase at this site. This has been noted in the context of chronic hepatitis C infection [65]. In addition, directly or indirectly, hepatitis C virus infection can modify MC behaviour. For example, MCs have been shown to upregulate HLA-G expression, associated with fibrosis, in HCV-infected livers or in response to IL-10 or type I IFN treatment. Increased MC expression of miRNA-490 has also been reported in response to HCV E2 protein [66]. Many reports have implicated MCs as drivers or contributors to profibrotic processes in tissues such as the liver, but their precise role remains unclear in this context. Similarly, mast cell activation could potentially help promote conditions necessary for tumor development, either through enhancing ongoing inflammatory processes or through promotion of early angiogenesis.

In addition to direct impacts of viral infection and viruses on MCs there are many indirect interactions which could lead to MC activation. An excellent example of this is the increase in circulating levels of protein Fv that occurs during hepatitis. Under normal circumstances protein Fv circulates at low levels, however, in the context of infection, these levels rise dramatically. In vitro, protein Fv was shown to activate human heart derived MCs inducing degranulation and lipid mediator production via interactions with the VH3 region of IgE [67,68].

## 7. Mast Cell Promotion of Effective Immunity and Response to Oncolytic Viruses

There is substantial evidence for increased numbers of MCs at the growing edge of several common tumor types. As described above, MCs act as sentinel cells for infection enhancing the earliest immune responses to pathogens and facilitating the development of subsequent immune responses. In this context, the ability of MCs to respond to viruses with a known propensity to infect and promote the immune response to tumors is of particular interest. However, very few studies have examined MC responses to the most well-established oncolytic viruses, such as HSV, VSV, and vaccinia virus. The role of MCs in oncolytic therapies is not well defined and would likely be highly dependent upon tissue site and tumor type. MCs also have key roles in the regulation of tumor angiogenesis making analysis of their roles complex. However, it is known that, in mouse models, MCs promote effective anti-HSV responses in the skin and eyes [69,70]. Similarly, MCs have been suggested to have site-protective roles against vaccinia virus infection at sites such as the skin [71,72]. These impacts may extend to promoting the types of immune responses that also combat tumors through the mobilization of effector cell populations or dendritic cell responses but could be highly dependent upon local MC density and other microenvironmental factors.

**Figure 1:**
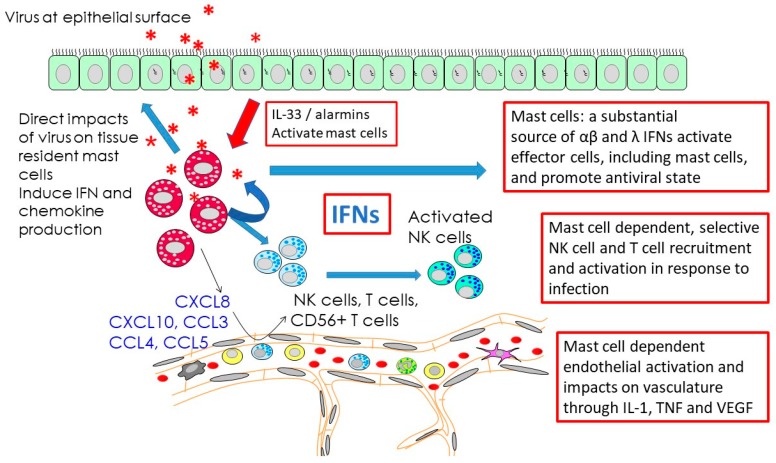
Selected, critical and early mast cell (MC) responses to viral infections which occur without a requirement for MC degranulation. MCs can be activated directly by active viral infection or by contact with viral particles. They can also be activated by alarmins released as a result of infection of neighboring or epithelial cells. This MC activation leads to the production of multiple mediators including large amounts of type I interferons (IFNs) and type III IFNs by virus infected human cells. While inducing an antiviral state in neighboring cells, such IFN responses also initiate multiple additional responses (blue arrows), the expression of a number of chemokines which together with MC-derived cytokines enhance the local recruitment of effector cells such as NK cells, T cells, and CD56-positive T cells from local blood vessels and promote the activation of NK cells, enhancing their cytotoxic functions. Such IFNs also act in an autocrine fashion to further promote selected mediator production by MCs. MC mediators, in several infections, would also act to enhance lymph node hypertrophy and mobilize local dendritic cell populations promoting the development of a subsequent acquired immune response.

The MC response to oncolytic reovirus, a common mucosal infection which has been used in clinical trials as an oncolytic treatment, is an excellent model for examining the role of MCs in an effective immune response to viral infection. Both human and mouse MCs can be productively infected with reovirus and this leads to a vigorous type I and type III IFN response. Infected MCs also produce substantial amounts of a range of chemokines. Of these, CXCL8 has been shown to be critical for the recruitment of human natural killer (NK) cells in response to viral infection both in vivo and in vitro [19,73], whereas, CD56-positive T cells are recruited by MC via a distinct mechanism [18]. The large amounts of type I IFNs produced by infected MC have the capacity to further activate recruited NK cells [19]. These interferons also act in an autocrine manner on MCs to promote both classical responses such as CXCL10 production and promote tissue remodelling and inflammation resolution through both VEGF and IL-1RA production [74]. Lytic viruses, such as reovirus and HSV, can also lead to the release of alarmins from cells; these include IL-33 which can activate MCs to produce a range of cytokines and chemokines [75] and promote neutrophil recruitment. Some key features of the degranulation-independent responses to reovirus and subsequent impacts of IFN and chemokine production, many of which are shared by other viral infection, are outlined in Figure 1. 

## 8. Pathogen Products that Activate Mast Cells

Over recent years, significant progress in MC research has uncovered the responses to pathogens and pathogen products from bacteria to viruses to fungi, and the mechanisms of activation. These studies have allowed us to better understand the roles of MCs in host defence against pathogens and serve as a foundation for developing improved therapeutics. Some key pathogen products that MCs can recognize are provided in Table 3, and some of the important interactions are described below. Given the increased propensity for bacterial and fungal infections associated with several viral infections and the widespread impact of pathogen products, beyond local sites of infection, it is important to consider both the impact of pathogen products, their mechanism of action, and direct pathogen–MC interactions to fully understand the role of these enigmatic cells in host defence and infectious disease pathology. We also need to consider that, especially in chronic or persistent infections, the ability of mast cells to mobilize acquired immune responses through actions on dendritic cells and promotion of T cell responses could lower the threshold for the development of autoimmune or chronic inflammatory disorders. In many cases, responses that do not usually, as single stimuli, involve degranulation such as those mediated by TLRs and RNA sensors, are similar in mechanism to those described for other cell types, especially monocytes, macrophages, and dendritic cells, although the predominant mediator responses may differ. However, the comprehensive innate immune receptor profile of mast cells together with their potent menu of preformed, newly-generated protein and lipid mediators ensures that they provide unique and rapid responses to many pathogen challenges. Notably, we need to recognize that responses to all forms of infection or pathogen challenge are complex and involve multiple cell types and microenvironmental signals. For this reason, it may be difficult to separate out the role of a specific receptor in the overall mast cell response. For example, during both viral and fungal disease mast cell degranulation in clinical settings has been reported, which is not normally observed following simple infection of mast cell in vitro or exposure of mast cells to pathogen products. Signaling through receptor systems, which may not induce degranulation alone, may contribute to this process by enhancing the expression or activity of receptors associated with degranulation such as complement receptors, other G-protein-coupled receptors or Fc receptors. Similarly, mast cell stabilizing agents, which have proven effectiveness in reducing IgE-mediated mast cell activation, may have limited effectiveness in regulating mast cell mediator release induced by the Fc receptor independent mechanisms typical of responses to pathogens. However, other immunosuppressive and anti-inflammatory agents, such as corticosteroids, may effectively modify or suppress aspects of the mast cell-pathogen response.

## 9. Bacterial Pathogens and Products

Bacterial pathogen products are well described to activate MCs through pattern recognition receptors (PRRs) located on the MC surface, which recognize and respond to pathogen-associated molecular patterns. Toll-like receptors (TLRs) can recognize a multitude of bacterial pathogen products. Peptidoglycan (PGN) is a key structural component of Gram-positive bacterial cell walls such as in the *Staphylococcus aureus* bacteria [109]. PGN from *S. aureus* has been well described to activate immune cells through TLR2-dependent mechanisms [110], and this has been shown in both murine and human MCs where activation led to increased production of inflammatory mediators GM-CSF and IL-1β [76,98]. TLR4-mediated responses are also important in MC-mediated host defence against Gram-negative bacteria such as *Escherichia coli*. It has been reported that activation of MCs by *E. coli*-derived LPS, through TLR4-dependent mechanisms, results in TNF and IL-6 proinflammatory cytokine production [77]. Similar to multiple viral stimuli, LPS activation of MCs has also been demonstrated to enhance IFN production by NK cells in vivo [111]. TLR9 detects CpG motifs enriched in bacteria and other micro-organisms [79]. MCs also respond to CpG-containing DNA activation through selective proinflammatory cytokine production. In addition to TLRs, MCs express a variety of Fc receptors. Immunoglobulin superantigens, such as protein A of *S. aureus,* can bind to immunoglobulins attached to FcϵRI on MCs. Activation of MCs through this mechanism by *S. aureus* protein A resulted in release of mediators such as histamine and leukotrienes [81,112]. Bacterial superantigens have also been reported to enhance MC activation, in some cases leading to degranulation, although impacts on cytokine production have been less well studied. Examples of these include enterotoxins A and B, and superantigen-like proteins (exotoxins) from *S. aureus* [82,83,84]. Bacterial toxins such as those derived from cholera, pertussis, and clostridium species have also been reported to be able to induce MC responses [85,86,87,113]. A wide variety of other more pathogen-specific interactions also occur. In vivo, complement activation also likely contributes to MC responses to bacterial products through MC receptors for C5a and C3a. As a result of expression of multiple receptors, MCs are well-equipped to detect and initiate a rapid response to bacteria and their pathogenic products either with or without concurrent degranulation. In most bacterial infections, multiple mechanisms of mast cell activation can be triggered through both direct pathogen interactions and indirect mechanisms.

## 10. Viral Pathogen Products

Viral products have been shown to activate MCs through multiple receptor types, as described above, and also through TLRs and other classical viral sensors. Double-stranded RNA (dsRNA) products of multiple viruses can activate MCs through TLR3 and other RNA sensors. Activation can result in increased type 1 interferons and recruitment of other immune cell types such as NK cells through chemokine production when stimulated with a viral dsRNA analog [73,90]. Other PRRs such as retinoic acid-induced gene I (RIG-1) can recognize and respond to intracellular viral RNA products such as dsRNA and uncapped viral RNA. Deficiency or knockdown of the RNA sensor RIG-1 in MCs resulted in blunted cytokine and chemokine production when challenged with influenza A virus and DENV, respectively [15,23,95]. The fundamental mechanisms by which mast cells respond to viral products are, in many cases, similar to those used by multiple other cell types. However, the ensuing mediator response is profound in the diversity of cytokines and chemokines produced and the amount and range of IFNs produced in several situations [16,18,19,38,73]. As described above for some bacterial pathogen products, viral pathogen products are able to activate MCs through Fc receptors found on the surface. These superantigens such as protein Fv (an endogenous protein produced by the liver during viral hepatitis) and envelope glycoprotein gp120 (human immunodeficiency virus type-1 (HIV-1) have been shown to bind to the V_H_3 region of IgE bound to FcϵRI on MCs, resulting in activation and release of different mediators [67,68,97]. Viruses can also produce a number of products that modulate immune activity. One of the best examples of this is Orf virus-encoded interleukin 10, such as that produced during Epstein Barr virus infection which has been demonstrated to enhance mast cell proliferation, similarly to mammalian IL-10 (see Table 3). These interactions are just some of the established mechanisms by which MCs are capable of recognizing viral pathogens and pathogen products to elicit appropriate immune responses. However, much more work needs to be done in this area to better define the nature and plasticity of MC responses to viral products.

## 11. Fungal Pathogens and Products

MCs are in a prime location to recognize foreign fungal pathogens and products to initiate host defence mechanisms. Complex interactions between MCs and fungal pathogens have been described, with both positive and negative regulatory roles involved. Zymosan, a cell wall component of *Saccharomyces cerevisiae*, has been shown to activate TLR2 signaling elements on MCs. Activation of TLR2 by zymosan led to increased proinflammatory mediator production such as GM-CSF and IL-1β as well as lipid mediator leukotriene C_4_ [98]. Apart from TLR activation by fungal pathogen products, other immune cell PRRs, such as dectin-1, have a major role in antifungal immunity through recognition and binding of β-glucans and zymosan as well [114,115]. MCs have been shown to express dectin-1, where signaling through this receptor by zymosan from *S. cerevisiae* resulted in increased reactive oxygen species and leukotriene C_4_ production [105,107]. Furthermore, *Candida albicans* yeast and hyphae resulted in MC degranulation, production of proinflammatory cytokines and chemokines, and recruitment of macrophages through increased chemotactic properties in a dectin-1-dependent manner [103,104]. Although limited in number, studies providing strong evidence regarding MC activation by fungal pathogens and products have been gaining recognition recently. For example, chitin and derivatives such as chitosan from fungal pathogens such as *Cryptococcus neoformans* have been shown to activate immune cells through pathways associated with TLR2 and Dectin-1 [100,101,102]. Furthermore, chitosan-containing nanoparticles have been shown to promote MC activation and IFN-gamma and IL-17 production associated with their adjuvant properties [116]. Mature fungal hyphae of *Aspergillus fumigatus* have also been reported to induce degranulation of MC with release of β-hexosaminidase [108]. A summary of major pathogen products inducing MC activation is provided in Table 3. 

## 12. Conclusions

**Figure 2:**
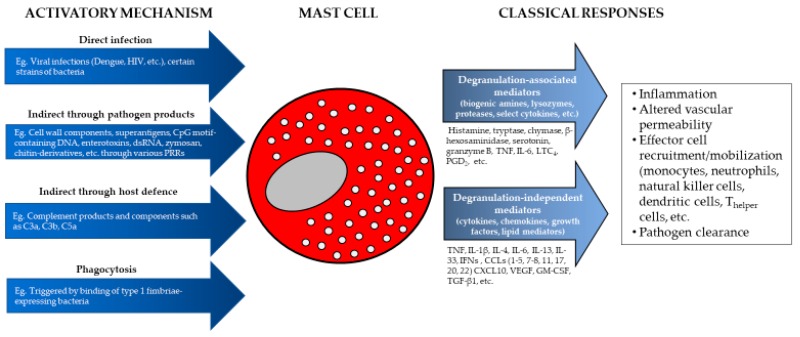
Mast cells can recognize and respond to pathogens either directly through pathogen infection, or indirectly through an array of pathogen products, host defense mechanisms, or phagocytosis. Activation results in the secretion of classical mast cell mediators that can be categorized as degranulation-dependent or degranulation-independent. These mediators contribute to the inflammation and changes to the site of pathogen infections, recruitment of other immune cell types, and regulation of the immune response to pathogens.

MCs are recognized as important participants in responses to viral infection and pathogen products in multiple settings, as summarized in Figure 2. Their impacts can be positive or negative, depending on the pathogen and immune status of the subject. However, we still have a relatively poor understanding of the scope and importance of MC responses in human disease. The substantial production of type I and II interferons by MCs in response to a number of viruses, together with their production of cytokines which activate endothelium and chemokines which promote effector cell recruitment allow for the rapid, local development of responses to pathogens. The impact of selected MC mediators on dendritic cells and local lymph nodes promotes longer term acquired immune responses. The ability of MCs to serve as sentinel cells during infection or tissue injury suggests that MC activation could be a relatively unexploited route to enhance immune responses in chronic infection or cancer. However, there are certainly situations where reducing some aspects of MC mediator production might be beneficial, such as in severe dengue disease. Key unanswered questions remain regarding the extent to which mast cells normally contribute to preventing infection and promoting effective immunity in human disease. We lack a proper understanding of many of the key signaling pathways involved in mediating selective cytokine and chemokine responses from mast cells. Since much of MC actions are at local sites of pathogen invasion, there may be opportunities to enhance effective early immunity at a local tissue level in sites such as the skin or airways. Too much of our current information is derived solely from rodent models and further work in a clinical setting to address these issues is urgently needed. Through a better understanding of the nature of MC responses to viruses and pathogen products and understanding how they are regulated, we can begin to build approaches to selectively activate these powerful immune cells to induce or inhibit local immune events and harness their functions therapeutically.

## Figures and Tables

**Table 1:** Key in vitro studies of mast cell responses to viral infection.

Human MC Source	Virus/Virus Replication	Degranulation	Lipid Mediator	Cytokine Synthesis	Additional Biological Responses	Citation
	**+ssRNA**					
HMC-1 cell line	RV16/yes	n.d.	n.d.	IL-6, IL-8, TNF-α, IFN-α	↑*ICAM* ↓ Cell viability	[10]
LAD cell line and CBMC	RV1B and RV16/yes	NO	n.d.	IFN-β and -λ; *CXCL10* and *CCL5*		[11]
HMC-1 and KU812 cell lines	RV14/yes	Enhanced following cross-linking of FcεRI	n.d.	Enhanced production of IL-8 and GM-CSF following cross-linking of the FcεRI	↑ ICAM	[12]
Skin MCs and human skin tissue	DENV type 2 (NGC and K0048)/yes	Yes	n.d.	CCL5, IL-6, IL-8, VEGF	-MC mediators releases in response to infection with DENV induce activation and proliferation of endothelial cells -DENV localized in MC cytoplasmic granules was shown to be infectious	[13]
HMC-1 and KU812 cell lines	DENV type 2 strain 16681/yes	n.d.	n.d.	n.d.	Anti-DENV neutralizing antibodies enhanced DENV infection in KU812 and HMC-1 cells in a mechanism involving autophagy	[14]
CBMC, HMC-1, and KU812	DENV type 2 strain 16681/n.d.	n.d.	n.d.	CCL2, CCL4, CXCL10, type I IFNs,	MC-derived type I IFNs prevented infection of KU812 with DENV	[15]
	**-ssRNA**					
	RSV long strain/limited	n.d.	No	CCL4, CCL5, CXCL10, IFN-α		CBMC [16]
HMC-1	RSV long strain/inefficient	Only in co-culture of MCs with RSV-infected A546 epithelial cells	n.d.	TNF-α only in co-culture of MCs with RSV-infected A546 epithelial cells		[17]
	**dsRNA**					
CBMC	Reovirus/yes	No	No	CXCL8, Type I IFNs, IL-10, TNF	Reovirus-infected MC induce the recruitment and activation of NK cells to sites of infection Recruitment of NKT [18] cells was also observed	[15,19]
Blood derived MC precursors	HIV-1 (M-tropic)/ yes	n.d	n.d.	n.d.		[20,21]
Non-human MCs						
P815 murine cell line	Influenza H1N1 (A/WSN/33), H5N1 (A/Chicken/Henan/1/04), H7N2 (A/Chicken/Hebei/2/02)/yes, dependent on MC apoptosis	n.d.	n.d.	IL-6, IL-18, TNF-α, and CCL2	MC apoptosis	[22]
Murine bone marrow MCs	Influenza Influenza H1N1 (A/WSN/33) virus/inefficient	Yes	Yes	CCL2, CCL3, CCL4, CCL5, CXCL2, CXCL9, CXCL10, IL-6, and TNF-α		[23]
P815 murine cell line	Influenza H5N1 (A/Chicken/Henan/1/04)	Yes	n.d.	IFN-γ		[24]
Porcine primary MCs	Influenza H1N1 (A/Ca/04/2009) virus/inefficient	Yes	n.d.	*IL1A, IL6, CXCL9, CXCL10, CXCL11*		[25]

**Table 2:** Key murine studies of MCs in viral infection.

Murine Model	Virus	Biological Responses Observed *	Implication(s)	Citation
Balb/c	Influenza H1N1 (A/PR/8/34)	Following infection, -MCs progenitors recruited to lungs -MCs associated with inflammatory cells surrounding bronchioles	Increased number of MCs in the lungs in response to influenza may be associated with virus-induced asthma exacerbations	[26]
Balb/c immunized with both the HA influenza protein and the MC activator C48/80	Influenza H1N1 (A/Ca/04/2009)	-Enhanced levels of serum IgG and mucosal IgA against HA protein. -Reduced levels of virus titers in lungs -Predominant Th1 over Th2 cellular responses	The vaccine approach combining HA and mucosal adjuvant C4/80 elicits protective immunity specifically [27] against H1N1 virus	[28]
C57BL/6 and B6.Cg-Kit^W-sh^	Influenza H1N1 (A/WSN/33)	MC-deficient mice -Less susceptible to lose weight -Showed reduced numbers of inflammatory cells in lungs	MCs are crucial effectors in the pathological innate immune responses	[23]
Balb/c	Influenza H5N1 (A/Chicken/Henan/1/04)	Severe bronchiolitis and infiltration of inflammatory cells to lungs were reduced in mice treated with ketotifen previous and during infection with H5N1 virus	MC activities, specifically degranulation, promote lung lesions during viral infection	[24]
C57BL/6NTac mice	DENV strain EDEN2	Many of the pathological changes derived from infection with dengue virus, including metabolic dysregulation and inflammation, were reversed by treatment of infected mice with ketotifen	Therapy for dengue virus infection may include the use of MC stabilizer drugs	[29]
C3H/HeN	DENV type 2 strain 16681	MC degranulation and production of CCL-2, CCL5, and CXCL10 in response to dengue virus infection were reduced in mice treated with antibodies targeting the NS1 dengue protein.	Dengue-associated pathological effects can be reduced using anti-NS1 antibodies by mechanisms involving inhibition of MC activities	[30]
C57BL/6 and MC-deficient Kit*^W-sh^*/ HNuhrJaeBsmJ	DENV type 2 strain 16681	Kit*^W-sh^* mice: -Were more susceptible to infection with DENV -Showed prolonged bleeding and enhanced production of macrophage-derived CCL2 and macrophage infiltration at inoculation sites	MCs and macrophages coordinately may restrict DENV infection in the skin	[31]
C57BL/6	DENV type 2, strain Eden 2	-MCs infected with DENV promote increased vascular permeability via chymase and leukotriene production -Usage of MC-stabilizing drugs restore vascular permeability in mice infected with DENV	-DENV-associated vascular leakage might be prevented by therapeutically targeting MC activities -Translation of these data to human settings showed chymase as a predictive biomarker distinguishing dengue fever from dengue hemorrhagic fever	[32]
C57BL/6	Vaccinia virus strain Western Reserve	-LAT-activated MCs showed improved antiviral activities against VV -MCs produce cathelicidin via TLR2 in response to LTA expressed by commensal bacteria	MCs primed via TLR2 fight more efficiently vaccinia virus	[33]

* Compared to control conditions.

**Table 3:** Major classes of direct mast cell responses to pathogen products.

Major Pathogen Products	Associated MC Receptor	Example of Pathogens	Citation
Bacterial Pathogens and Products			
Peptidoglycan	TLR2	*S. aureus*	[76]
Lipopolysaccharide	TLR4	*E. coli*	[77]
CpG motif-containing bacterial DNA	TLR9 ^†^	Multiple strains	[78,79]
Fimbriated adhesion molecule H	CD48	Fimbriated *E. coli*	[80]
Protein A	Fc receptors	*S. aureus*	[81]
Staphylococcal enterotoxins	Undefined	*S. aureus*	[82,83]
Staphylococcal superantigen-like proteins	TLR2	*S. aureus*	[84]
Cytolysin	Substance P receptor	*V. cholerae*	[85]
Pertussis toxin	CD48	*B. pertussis*	[86]
Clostridium toxin		*C. difficile*	[87]
Mycobacterial antigens		*M. tuberculosis*	[88,89]
Viral Pathogens and Products			
dsRNA	TLR3	RSV, Reovirus	[73,90]
ssRNA	TLR7 ^†^	Influenza A, VSV, Sendai	[78,91]
CpG motif-containing viral DNA	TLR9 ^†^	mCMV, HSV	[78,92,93,94]
dsRNA, uncapped viral RNA	RIG-I	Influenza A, Dengue	[15,23,95]
Orf virus-encoded IL-10	IL-10 receptor	Epstein Barr virus	[96]
Superantigens (Protein Fv, envelope glycoprotein gp120)	Fc receptors	Viral hepatitis, HIV-1	[67,68,97]
Fungal Pathogens and Products			
Yeast zymosan, chitin and derivatives ^†^	TLR2	*C. albicans S. cerevisiae, C. neoformans*	[98,99,100,101,102]
β-glucans, zymosan, chitin and derivatives ^†^	Dectin-1	*C. albicans*, *S. cerevisiae, C. neoformans*	[99,100,101,103,104,105,106,107]
Mature fungal hyphae	IgE-independent; StuA and MedA transcription factor-mediated	*Aspergillus fumigatus*	[108]

^†^ Inferred from current studies on other immune cells, but not directly demonstrated.
